# EX-PRESS Glaucoma Filtration Device: Management of Complications

**DOI:** 10.3390/vision4030039

**Published:** 2020-08-16

**Authors:** Michele Nicolai, Alessandro Franceschi, Paolo Pelliccioni, Vittorio Pirani, Cesare Mariotti

**Affiliations:** Eye Clinic, Polytechnic University of Marche, via Conca 61, 60126 Ancona, Italy; michele.nicolai@hotmail.it (M.N.); paopel@hotmail.it (P.P.); piranivittorio@virgilio.it (V.P.); mariottiocul@gmail.com (C.M.)

**Keywords:** glaucoma surgery, EX-PRESS, complications

## Abstract

The EX-PRESS glaucoma filtration device appears to be an effective addition to our options to treat refractory glaucoma. The possibility to create a sclerostomy without tissue excision provides a safe and reliable outflow pathway for aqueous that is standard in size, reducing much of the variability associated with a surgical procedure. Prospective randomized studies comparing EX-PRESS implantation with trabeculectomy show encouraging results. However, complications usually encountered in filtration surgery have been reported, and EX-PRESS implantation can also lead to specific device-related complications. This article reviews the most common complications associated with this procedure.

## 1. Introduction

The EX-PRESS glaucoma filtration device (Alcon Laboratories Inc., Fort Worth, TX, USA) has gained increasing recognition as an alternative to trabeculectomy. The shunt consists in a non-valved biocompatible stainless steel device 2.64–2.96 mm long with a 400 micron external lumen diameter and options of a 50 micron or 200 micron internal lumen diameter. The device connects the anterior chamber to the intrascleral space to divert the aqueous humor to the subconjunctival space, with aqueous shunted from the anterior chamber to beneath a partial thickness scleral flap. Mitomycin C (MMC) use at a concentration of 0.2–0.4 mg/mL during the procedure is recommended to prevent scarring. MMC can be applied to tissues using surgical sponges, while an alternative method involves the irrigation of MMC directly in the subtenon space; the different MMC application route may lead to changes in surgical outcomes [1].

The shunt is available in different versions with an inner lumen of 50 μm (R-50, P-50) and 200 μm (P-200). Originally, the EX-PRESS shunt was implanted under the conjunctiva, but this unguarded technique resulted in several complications such as hypotony and extrusion and erosion of the implant [2,3,4,5]. To avoid this complication, Dahan and Carmichael proposed a new technique to implant the device under a 5 × 5 mm partial-thickness scleral flap [6]. This safer method has completely replaced the original technique and has obtained encouraging results; numerous publications have confirmed the safety and efficacy of the device [7,8,9]. The procedure is similar to trabeculectomy but offers the advantages of a standard stented outflow route that eliminates the need of sclerostomy and peripheral iridectomy. The purpose of this review is to outline the most common complications of this procedure.

## 2. Failure

Similarly to all filtration surgeries, failure caused by episcleral and subconjunctival fibrosis remains a challenging problem. Although success criteria definition may differ between studies, many comparisons between EX-PRESS and standard trabeculectomy showed similar failure rates [10,11,12,13].

A recent meta-analysis based on four randomized controlled trials of 292 eyes confirmed that EX-PRESS implantation and trabeculectomy have similar efficacy in intraocular pressure (IOP)-lowering, medication reduction, vision recovery, and qualified operative success rates. EX-PRESS implantation is associated with higher rates of complete operative success and fewer hyphemia cases when compared to trabeculectomy [14].

In our series of 248 consecutive patients with uncontrolled glaucoma who underwent EX-PRESS implantation over a period of 9 years (with a maximum follow-up of 7 years), we found a complete success rate decline from 83% at 1 year to 57% at 5 years [9]. Similarly to trabeculectomy, the main risk factors for failure when using the EX-PRESS are diabetes, non-Caucasian race, and previous glaucoma surgery, probably due to excessive scar formation/high transforming growth factors (TGFs) concentrations [9].

Another recent study evaluating Japanese patients found that advanced age, higher postoperative IOP, pseudo-exfoliation glaucoma, and simultaneous cataract surgery negatively influence the volume of the filtration bleb 2 years after surgery [15]. It must be said that in the latest study, hemoglobin-A1C was not measured, and EX-PRESS represented the first-line surgical treatment, without previous or subsequent glaucoma surgeries; therefore, it is difficult to make direct comparisons between our study and the Japanese report.

Failure of EX-PRESS shunts implanted under a scleral flap is normally a consequence of episcleral or bleb scarring, despite the use of anti-metabolites. Growth of fibrotic tissue between the external flange of the device and the inner portion of the scleral flap has been shown in vivo [16] and in enucleated eyes [17], representing a possible mechanism of failure.

The first option to consider in case of failure is a bleb needling or revision with antifibrotic agents, which have been proven to be a safe and efficacious procedure in eyes with an EX-PRESS shunt [18]. Another recently reported ab externo approach involved the use of an extendible 41-G subretinal injection needle, to quickly restore device patency when the conventional bleb revision was not effective [19]. The needle has a diameter of about 100 μm, and it is composed of Teflon; therefore, it does not allow cannulating devices with the lumen of 50 μm. This procedure could be used when Nd:YAG laser fails to restore the flow [19]. However, more reports are needed to determine the efficacy of this new approach.

In case of definitive failure, the EX-PRESS shunt does not need to be removed to proceed to additional glaucoma surgery. Conventional tube surgery can be performed avoiding the area of the failed bleb.

## 3. Hypotony

Most studies show no significant difference in intraoperative or postoperative complications, but some do note a lower incidence of postoperative hypotony and choroidal effusion with use of the EX-PRESS [10,11,20,21].

This finding might be related to the smaller and standard outflow pathway created by insertion of the shunt. However, postoperative hypotony has been reported after surgery, with a transient decrease in the anterior chamber depth, probably due to a transient overfiltration through the EX-PRESS implant [22].

Nonetheless, management of postoperative hypotony can be achieved in the same manner as in the classic filtration procedures.

A significant reduction in the axial length, with an important hyperopic refractive shift, was recently observed in a highly myopic patient 18 months following hypotonia after glaucoma filtering surgery with EX-PRESS [23].

EX-PRESS shunt malposition

The EX-PRESS shunt is designed with a flange that fixes the implant to the sclera and prevents it from penetrating the anterior chamber. It also has a small spur-like extension to prevent movement extrusion.

To avoid shunt movement after the insertion, it is essential to perform the pre-incision with the correct needle size. A 25-gauge needle track for the newer P series shunts is required, because of the slightly squared off shape. EX-PRESS glaucoma shunt dislocation into the anterior chamber has been described by Teng et al. twenty-one days after the procedure with no evidence of corneal decompensation [24]. Moreover, Song et al. recently reported a case of impending extrusion, due to previous improper spur fixation, treated by shunt-position adjustment [25]. Vetrugno et al. published a technique to secure the EX-PRESS flange knotting its flange to scleral bed with a polyprolene suture to ensure a correct repositioning after dislocation of the device [26]. A misplaced needle-track or a shunt migration can result in implant–iris touch. If the tip of the tube deeply indents the iris, it is advisable to remove the device as it may cause cataract formation. This complication was more frequent with the original model, R50, because of its longer tube. Traumatic cataract caused by the EX-PRESS has also been shown in a patient with aniridia [27]. It is therefore advisable to avoid EX-PRESS implant in narrow angle cases without lens extraction. Malposition of an EX-PRESS shunt might also lead to corneal inflammation, resulting in acquired corneal neuropathy and photoallodynia [28].

## 4. Erosion

The original technique of implantation under a conjunctival flap was associated with high rates of implant dislocation and subsequent erosion [29].

Traverso et al. showed, in a series of 26 eyes of 25 patients using the sub-conjunctival technique, two cases (7.7%) of device rotation (one treated by reposition) and three cases (11.5%) of conjunctival erosion at 2 and 3 years [30]. Erosion and subsequent exposure often require EX-PRESS removal.

To remove the device, after an accurate dissection of the overlying conjunctiva and identification of the scleral flap, any fibrotic tissue should be dissected to allow for lifting of the flap. Once the shaft is fully exposed, the device should be gently lifted and rotated to dislodge the distal spur and allow removal. Creating a wider incision for removal is not needed to perform a safe extraction of the shunt.

If the integrity of the scleral flap allows a tight closure, a tissue punch can be used to further enlarge the original entry site and convert to traditional trabeculectomy. If the scleral flap has eroded significantly, a patch graft should be used to close the opening and secured tightly to prevent filtration through this site.

## 5. Shunt Occlusion

The EX-PRESS glaucoma filtration device distal portion contains an accessory orifice which serves as an alternative route in case of obstruction of the main central lumen. In a case series of 345 EX-PRESS shunt implantations, Kanner et al. reported that the most common device-related complication was shunt obstruction (six eyes, 1.6%) [7]. Only one of the six shunts was blocked with vitreous, but no visible obstruction was observed in the others. In this circumstance, Nd:YAG laser treatment of the shunt tip appears to be a safe and effective procedure to restore shunt drainage [31]. It has also been shown that transient shallowing of the anterior chamber can cause the development of synechiae or fibrin membrane around the shunt and obstruct the axial port and, eventually, the accessory port [32].

## 6. Corneal Decompensation

It has already been shown that the application of MMC may play a role in the pathogenesis of bullous keratopathy after filtrating surgery [33,34]. There are still limited data in the literature about the effect of EX-PRESS on endothelial cellular density.

In the first study that has compared endothelial changes after trabeculectomy, EX-PRESS shunt and Ahmed valve implantation, Casini et al. did not find significant modifications either at 1 month (0.2% decrease) or at 3 months (0.3% decrease), suggesting that EX-PRESS might represent the treatment of choice in patients with significant low corneal endothelial cell density before surgery or other risk factors for corneal damage [35]. The lower loss of corneal endothelial cellular density with EX-PRESS implantation might reflect endothelial protection from the lack of over-filtration in the early postoperative period. EX-PRESS implantation combined with phacoemulsification cataract extraction has been proven to be a safe and effective technique for reducing IOP and antiglaucoma medications in patients with cataract, with rare device-related complications on long-term follow-up even in primary angle closure glaucoma [36,37].

Recent studies evaluating longer follow-ups demonstrate a statistically significant reduction of the corneal endothelium cell density, which was found by Ishida et al. 24 months after EX-PRESS implantation [38], compared to the normal corneal endothelium decrease rate of 0.6% per year [39]. While comparing postoperative complications between trabeculectomy and EX-PRESS implantation with 2 years of follow-up, Arimura et al. showed that EX-PRESS is associated with a significantly greater reduction of corneal endothelial cell density than trabeculectomy, especially in the corneal area around the tube [40].

On the other hand, Omatsu et al. found no effects on the corneal endothelial cells until at least 24 months after the initial EX-PRESS surgery, supporting the benefit of using this procedure in patients who have a lower corneal endothelial cell density prior to the surgery [41].

The physiopathological mechanism underlying the damage to the corneal endothelial cells following EX-PRESS implantation remains unknown, with recent data suggesting that it might be different from that of corneal endothelial cell loss after trabeculectomy. The corneal superior area, where the EX-PRESS device was inserted, was found to have significantly more severe corneal endothelial cellular density reduction than the inferior area 2 years after surgery, whereas no area dependency was observed in patients who underwent trabeculectomy [40].

Possible reported causes of endothelial cell loss after EX-PRESS are MMC (although pathophysiology is still not clear), foreign body reaction or immune reactions due to the stainless material used for the tube, changed flow of the aqueous humor around the tip of the tube, and mechanical ablation between the corneal endothelium and the tube.

To reduce the risk of a postoperative contact between the tube and the endothelium, the insertion of the device should be performed while the anterior chamber is inflated by a continuous infusion or secured with viscoelastic. The needle entry point is at the grey line (junction between sclera and cornea) to avoid corneal stroma. Tojo et al. reported a case of partial decompensation following filtering surgery with EX-PRESS of the corneal endothelium adjacent to the filtering bleb 9 months after the surgery. Specular microscopy also revealed a marked reduction in the endothelial cell density at the center of the cornea. However, the authors underlined that the device was erroneously inserted from the cornea, not from the trabecular meshwork [42].

Corneal surgery (with Descemet membrane endothelial keratoplasty [DMEK] or Descemet stripping automated endothelial keratoplasty [DSAEK]) may be taken into consideration in cases of severe endothelial cell loss, and patients presenting lower endothelial cell count should be carefully evaluated and informed before EX-PRESS implant surgery.

## 7. Infection

Trabeculectomy bleb-associated infections are a serious and vision-threatening complication whose incidence varies among different studies; however, there is cumulative evidence that the incidence of bleb-associated infections has reduced over time [43]. The Collaborative Initial Glaucoma Treatment Study (CIGTS) found a 5-year risk of blebitis and bleb-associated endophthalmitis of 1.5% and 1.1%, respectively, after trabeculectomy [44].

While evaluating longer follow-ups, a paper on 460 eyes which underwent trabeculectomy reported cumulative probabilities of blebitis and bleb-associated endophthalmitis of 2% and 5%, respectively, during the 20 postoperative years [45].

Little has been published regarding the incidence of blebitis and endophthalmitis after EX-PRESS implantation. Few cases of bleb-related endophthalmitis following EX-PRESS implantation have been reported [46,47], including one in which there was formation of a corneal infiltrate overlying the EX-PRESS device, which resolved with corneal scarring after device removal [48]. The management of blebitis in the presence of an EX-PRESS shunt is controversial. Some authors advocate similar treatment to that for blebitis after trabeculectomy [46], while others recommend device removal [3].

## 8. Conclusions

The aim of every glaucoma surgeon is to minimize complications while maximizing outcomes. The EX-PRESS glaucoma filtration device appears to be an effective addition to our surgical options to lower IOP in patients with refractory glaucoma. The possibility to create a standard sclerostomy without tissue excision provides a safe outflow pathway for aqueous with a standard size, reducing much of the variability associated. Prospective randomized studies comparing EX-PRESS implantation with standard trabeculectomy show encouraging results. However, the usual complications encountered in filtration surgery have been shown, and the implant of the EX-PRESS can also lead to specific device-related complications. The correct positioning of the implant is crucial for preventing these complications.
